# Transcriptomic Markers of Immunosenescence in Cynomolgus Macaques: A Pilot Study

**DOI:** 10.3390/genes17080944

**Published:** 2026-08-13

**Authors:** Viktoria M. Petrova, Dmitry V. Bulgin, Elena Yu. Radomskaya, Vsevolod A. Shevelov, Darya S. Zhukova, Olga. P. Chzhu, Andrey D. Manakhov, Alexander V. Popov, Stanislav A. Rybtsov

**Affiliations:** 1 Research Center for Genetics and Life Sciences, Sirius University of Science and Technology, Sirius 354340, Russia; petrova.vm@talantiuspeh.ru (V.M.P.);; 2 Kurchatov Medical Primatology Center, National Research Center ”Kurchatov Institute”, Sochi 354376, Russiazoomlenazoom@gmail.com (E.Y.R.); popov@neuro.nnov.ru (A.V.P.); 3 Department of Genomics and Human Genetics, Vavilov Institute of General Genetics, Russian Academy of Sciences, Moscow 119991, Russia

**Keywords:** *Macaca fascicularis*, cynomolgus macaques, aging, transcriptome, bone marrow, blood, immunosenescence

## Abstract

Background: One of the key hallmarks of aging is the age-related decline in immune system function, accompanied by a chronic low-grade inflammation, or “inflammaging”. Simultaneously, a reduced capacity of immune cells to recognize and eliminate pathogens, along with immune exhaustion, is also defined as a sign of aging. Cynomolgus macaques (*Macaca fascicularis*) belong to a group of non-human primates evolutionarily close to humans and are often used for preclinical research. Methods: In this study, we performed mRNA sequencing of bone marrow and peripheral blood samples from young (5 years old) and old (over 19–21 years old) cynomolgus macaques to identify key markers of immunosenescence. Results: Although an increase in p16 expression was detected, we did not observe the increase in the senescence-associated secretory phenotype (SASP) cytokines reported in previous studies. Instead, we observed a transcriptional profile characterized by increased lymphocyte cytotoxic activity combined with a decrease in proinflammatory signaling, reduced markers of myeloid cells, and lowered sensitivity to pathogen-associated patterns. Similar changes were detected in both blood and bone marrow: decreased expression of naive T-cell markers (*CCR7*, *LEF1*, *SELL*, and *FOXO1*), reduced markers of the myeloid lineage—neutrophils and monocytes (*CD177*, *CD14*, *CD163*, *FPR1*, *FPR2*, and *CXCR1*)—and downregulation of genes belonging to different pattern-recognition receptor families (*TLR1*, *TLR2*, *TLR4*, *TLR5*, *TLR6*, *TLR8*, *TLR10*, *IFIH1*, *CLEC4E*, *NOD2*, *NLRC4*, *NLRP12*, *NLRX1*, and *NAIP*). In contrast, the group of old animals showed increased expression of markers associated with terminally differentiated cytotoxic lymphocytes (CD8+ T cells and NK cells): *GZMB*, *PRF1*, *KLRK1*, *FASLG*, *TBX21*, *CCR5*, and *GNLY*. Conclusions: Our findings offer new perspectives on the molecular mechanisms of age-associated immune dysregulation in non-human primates, serving as a baseline for selecting key candidate genes in subsequent functional investigations.

## 1. Introduction

Cynomolgus macaques (*Macaca fascicularis*) are Old World non-human primates widely used in biomedical research due to their phylogenetic proximity to humans, physiological similarities, compact size, and suitability for controlled housing [1,2,3]. While shorter-lived model organisms, including rodents, zebrafish, and invertebrates, have provided fundamental insights into conserved mechanisms of aging, such as insulin/IGF-1 signaling and aberrant RNA processing [4], these models differ substantially from humans in immune system architecture, lifespan, and overall physiological context. In contrast, the cynomolgus macaque represents an important translational bridge in gerontology, providing a clinically relevant preclinical model for investigating the complex biology of human aging, which is driven by the progressive accumulation of genetic and epigenetic alterations [5], and for evaluating the efficacy and safety of candidate geroprotective interventions.

The immune system of the cynomolgus macaque closely resembles that of humans, sharing key features such as the complexity of T- and B-cell subsets, major histocompatibility complex (MHC) genetics, and age-associated immune remodeling. Consistent with human aging, cynomolgus macaques exhibit hallmark features of immune system aging, including a progressive decline in naive T-cell frequencies, accumulation of terminally differentiated memory T cells, diminished vaccine responsiveness, and elevated circulating levels of proinflammatory cytokines [3,6,7,8,9,10,11,12].

Immune system aging is a complex process affecting primary (bone marrow and thymus), secondary (spleen and lymph nodes), and tertiary lymphoid tissues. Age-related structural disorganization within these niches reduces the release of bone marrow-derived precursors and impairs the maturation of naive cells into specialized effector lineages. Furthermore, age-related thymic involution compromises the production of naive T cells [13,14,15,16]. As a result, memory T and B cells become dominant during immune responses; over time, individual dominant T- and B-cell clones gradually displace the broader memory pool, ultimately narrowing the T-cell receptor (TCR) and B-cell receptor (BCR) epitope recognition repertoires [15,17,18,19,20,21].

In parallel with the degradation of the T-cell compartment, systemic inflammatory processes emerge with age, exacerbating lymphocyte dysfunction. This complex of age-associated changes is accompanied by the accumulation of senescent immune cells with an altered secretory profile [22]. These cells interfere with the proliferative capacity of T and B lymphocytes and further contribute to the narrowing of the TCR and BCR repertoires [20,21,23]. As a result, cooperation between immunocompetent cells is disrupted, ultimately impairing the formation of a robust adaptive immune response.

Age-related changes in innate immunity are multifaceted, encompassing a diverse array of cellular mechanisms. Increasingly, aging is conceptualized as being driven by the accumulation of senescent cells, which secrete a complex profile of factors known as the senescence-associated secretory phenotype (SASP). This inflammatory cocktail typically includes TNF, IL-1β, IL-6, and IL-8 (CXCL8), among others. The accumulation of these cells is directly linked to the development of age-related chronic, low-grade inflammation, commonly termed “inflammaging” [24,25,26,27].

With advancing age, Toll-like receptor (TLR) signaling undergoes significant disruption: the capacity of TLRs to recognize pathogen-associated molecular patterns (PAMPs) declines [28,29,30], and the production of type I interferons by dendritic cells (DCs) is characteristically suppressed [31,32]. Concurrently, functional exhaustion of the innate immune system manifests as reduced phagocytic activity in neutrophils [27,33,34], although some studies suggest that myeloid cell function remains relatively stable in both humans and non-human primates [35]. Extensive transcriptome profiling in rodent models has linked bone marrow aging to the upregulation of proinflammatory factors, which contribute to the depletion of the hematopoietic stem and progenitor cell (HSPC) pool. For example, the detrimental effects of TNF and IL-6 have been shown to drive this decline in the HSPC pool [36,37,38]; however, such findings remain insufficient and frequently contradictory in primate models [8,35,39,40].

In this study, key transcriptomic markers of immunosenescence in the bone marrow and peripheral blood of aged cynomolgus macaques (>20 years) were identified through comparison with a control group of young animals (~5 years). These findings provide new insights into the mechanisms underlying age-related bone marrow decline and elucidate the core aging patterns common to both bone marrow and peripheral blood.

## 2. Materials and Methods

### 2.1. Animals and Housing Conditions

The animals were maintained at the Kurchatov Medical Primatology Center of the National Research Center “Kurchatov Institute”. Two groups of sexually mature, healthy male cynomolgus macaques (*M. fascicularis*) were selected for the study: young animals (*n* = 3, aged 5 years, corresponding to approximately 17–18 human years) and old animals (*n* = 3, aged 19, 20, and 21 years, corresponding to approximately 66–70 years of human biological age). All animals were originally imported from Southeast Asia and bred for 5–6 generations within the Center for Medical Primatology. Animals included in this study were not genetically related in at least two previous generations.

The macaques were housed in an open pen connected to enclosed rooms maintained at controlled temperatures of 23–28 °C and approximately 70% relative humidity under natural daylight conditions. The animals were housed in age-matched male groups and had access to both the open-pen and enclosed-room environments. Two weeks before sampling, they were separated into individual rooms under the same control conditions (temperature of 23–28 °C, humidity of ~70%, and natural daylight).

The animals received a balanced diet containing sufficient protein, fat, carbohydrates, vitamins, and dietary fiber (vegetables, fruits, pelleted feed, and biscuits). Access to fresh drinking water was provided ad libitum via a centralized supply system in compliance with state sanitary standards (SanPiN 2.1.3684-21, SanPiN 1.2.3685-21; Rospotrebnadzor, Moscow, Russia) [41,42]. All experimental procedures were conducted in compliance with the recommendations of the International Association for Assessment and Accreditation of Laboratory Animal Care (AAALAC). The animal study protocol was approved by the ethical permission of the Kurchatov Center of Medical Primatology (No. 02-3pr, dated 21 March 2024). All macaques included in the study were confirmed healthy by routine veterinary examinations, negative screening for tuberculosis and herpes B virus, and a review of prior medical records. Detailed individual metadata for all animals included in this study are provided in Table S1.

### 2.2. Sample Collection

The sampling procedure was performed sequentially on the same day for all animals by two qualified veterinarians. To achieve balanced, soft chemical fixation, 1.7 mg/kg Vesotil (tiletamine and zolazepam) was used in combination with 2 mg/kg Xyla (xylazine) at a lower dose. This combination provides rapid sedation, muscle relaxation, and analgesia in monkeys. The medication was administered by intramuscular injection into the gluteal muscle. Twenty to thirty minutes after the disappearance of reflex responses, veterinarians sequentially collected blood and bone marrow samples. The animals’ heart rate and respiratory activity were monitored throughout the procedure. Peripheral blood samples were collected first from the superficial femoral vein, and then using a Jamshidi needle, one milliliter of bone marrow was aspirated from the upper third of the tibial shaft (Figure S1). The cellular material was transferred to tubes containing heparin and phosphate-buffered saline (PBS). The material was resuspended by gentle pipetting. After assessing the number of nucleated cells, the required aliquot was immediately removed for subsequent RNA extraction. The protocol for bone marrow extraction in non-human primates was adapted at the Scientific and Practical Center for Pathological Anatomy of Laboratory Animals, part of the Center for Medical Primatology.

Total counts of leukocytes, granulocytes, monocytes, and lymphocytes were immediately measured in whole blood using a MEK-7300K automated hematology analyzer (Nihon Kohden, Tokyo, Japan). To lyse erythrocytes, 10 volumes of BD Pharm Lyse^TM^ lysis buffer were added to the cell pellet, followed by a 10 min incubation at room temperature. The cells were washed once with PBS by centrifugation at 300× *g* for 10 min. The resulting cell pellet was resuspended in PBS/2% FCS, counted, and immediately processed for RNA extraction.

Part of the study was performed using equipment at the “Primat” Core Facility of the Kurchatov Center of Medical Primatology. This work was conducted as part of a state assignment to the National Research Center “Kurchatov Institute”.

### 2.3. RNA Isolation and Quality Control

A cell pellet of approximately 1 × 10^6^ cells was homogenized in 1 mL of ExtractRNA reagent (Evrogen, Moscow, Russia). RNA extraction was performed according to the manufacturer’s protocol. RNA quantity was assessed using a NanoDrop OneC microvolume UV spectrophotometer (Thermo Fisher Scientific, Waltham, MA, USA). Genomic DNA contamination was removed using DNase I (RNase-free, 1 U/μL) according to the manufacturer’s instructions (Thermo Fisher Scientific, Waltham, MA, USA). The concentration of DNA-free RNA was measured using a Qubit 4 fluorometer (Thermo Fisher Scientific, Waltham, MA, USA). RNA integrity was determined by calculating the RNA integrity number (RIN) via capillary gel electrophoresis on a TapeStation 4150 system (Agilent Technologies, Santa Clara, CA, USA).

### 2.4. Library Preparation and Sequencing

High-quality RNA samples (RIN: 7.7–9.1) were selected for transcriptome library preparation (Table S2). mRNA was isolated using the NEBNext^®^ Poly(A) mRNA Magnetic Isolation Module, and directional libraries were prepared using the NEBNext^®^ Ultra^TM^ II Directional RNA Library Prep Kit for Illumina according to the manufacturer’s protocol (New England Biolabs, Inc., Ipswich, MA, USA). Library molarity was quantified via qPCR using the VAHTS Library Quantification Kit for Illumina 2.0 (Vazyme, Nanjing, China). Sequencing was performed in a paired-end 2 × 151 bp mode on a NovaSeq 6000 system (Illumina, San Diego, CA, USA).

### 2.5. Data Processing

More than 30 million reads were obtained for each sample. Raw read quality control was performed using FastQC v0.11.9 [43]. Adapters and low-quality reads were trimmed using AdapterRemoval v2.3.3 [44]. The filtered reads were aligned to the *M. fascicularis* reference genome (GCF_037993035.2) using HISAT2 v2.2.1 [45]. The number of reads mapped to gene exons was quantified using featureCounts v2.0.3 [46] based on the *M. fascicularis* genome annotation (GCF_037993035.2). RNA-seq quality control was performed using the CollectRnaSeqMetrics tool from Picard v2.26.11 [47]. All samples demonstrated high quality, with mRNA enrichment exceeding 76.2% (PCT_MRNA_BASES), highly efficient strand specificity above 97.6% (PCT_CORRECT_STRAND_READS), uniform transcript coverage across the bodies of genes (MEDIAN_CV_COVERAGE = 0.39–0.48) and ribosomal RNA contamination ranging from 0.08% to 2.25% (PCT_RIBOSOMAL_BASES). A detailed summary of all quality control metrics is provided in Table S2.

### 2.6. Differential Gene Expression Analysis

Downstream statistical analysis was performed in the RStudio environment using R v4.5.3. Read counts mapping to exons were normalized, and differential expression analysis comparing the old macaque group against the young macaque control group was conducted using the DESeq2 v1.50.2 package [48]. The analysis was performed independently for bone marrow and peripheral blood samples. Genes were considered differentially expressed (DEGs) if they met the thresholds of |log2FC| > 1 and a Benjamini–Hochberg adjusted *p*-value (*p*.adj) < 0.05.

### 2.7. Functional Enrichment Analysis

To facilitate functional interpretation, *M. fascicularis* genes were converted to their corresponding human orthologs using the biomaRt v2.66.2 package [49]. Gene Ontology biological process (GO BP) enrichment analysis was conducted separately for each tissue using the clusterProfiler v4.18.4 package [50] with pvalueCutoff = 0.05 and qvalueCutoff = 0.05 thresholds. Functional interpretation of the entire expression profile was performed via Gene Set Enrichment Analysis (GSEA) against GO BP [51] and KEGG (Kyoto Encyclopedia of Genes and Genomes) [52] databases using clusterProfiler v4.18.4 [50] with a *p*.adj threshold of 0.05 (pvalueCutoff = 0.05). To visualize relationships between enriched terms and identify functional clusters based on the top 10 most significant GO terms, enrichment map plots were generated using the emapplot function from the enrichplot v1.30.5 package [53]. To identify shared transcriptional patterns, up- and downregulated DEGs from bone marrow and peripheral blood were intersected. Functional enrichment analysis of the overlapping gene sets was performed using the Metascape web resource [54]. Individual gene expression box plots were generated using the ggplot2 v4.0.3 package [55].

## 3. Results

### 3.1. Individual Transcriptomic Changes and Biological Processes in Bone Marrow and Blood During Aging

Comparison of the bone marrow transcriptome profiles between young and aged cynomolgus macaques revealed 1471 DEGs. Of these, 769 genes were upregulated and 702 were downregulated in the old cohort (Tables S3 and S4, Figure S2A). Analysis of peripheral blood revealed 4137 DEGs, with 2024 genes upregulated and 2113 downregulated in aged animals (Tables S5 and S6, Figure S2B). Heatmaps depicting the top 50 significant DEGs in bone marrow (Figure S2C) and peripheral blood (Figure S2D) revealed two distinct gene clusters with opposing expression dynamics across age groups.

Principal component analysis (PCA) for bone marrow (Figure S2E) and blood (Figure S2F) demonstrated that, based on the overall gene expression profiles, the samples clustered into two distinct groups corresponding to the young (green) and old (red) macaques. Notably, while samples from young individuals were tightly clustered, those from the aged cohort exhibited substantial dispersion along the second principal component (PC2). Examination of the third principal component (PC3), which explained 13.6% and 9.6% of the variance in bone marrow and blood, respectively, did not reveal any additional age-related structure (Figure S3). Together with the dispersion along PC2, this observation suggests that the latter principal components primarily reflect inter-individual biological variability, including environmental influences such as a history of pathogen exposure.

**Figure 1:**
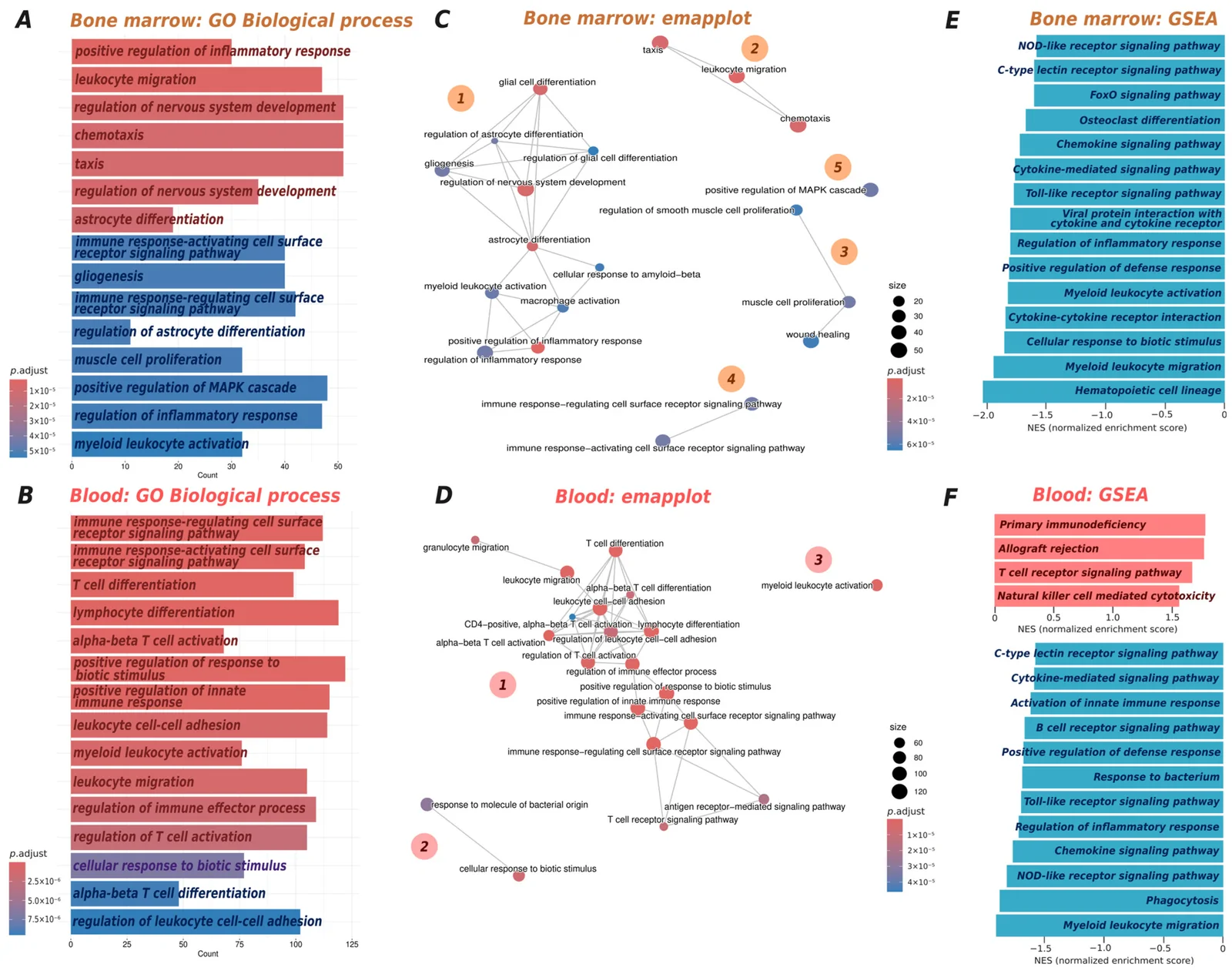
(**A**,**B**) GO biological process enrichment analysis (GO BP) in bone marrow (**A**) and blood (**B**). The 15 most significant biological processes for each tissue are shown. Bar length corresponds to the number of DEGs (count) included in the biological process, and the color scale reflects the significance level (*p*.adjust). (**C**,**D**) Enrichment maps (emapplots) of the top 20 significant GO biological processes in bone marrow (**C**) and blood (**D**). The color bar indicates the significance level (*p*.adjust); the circle size represents the number of genes involved in each biological process. (**E**,**F**) GSEA of selected immune-associated gene sets in bone marrow (**E**) and blood (**F**). Bars represent normalized enrichment scores (NES). Red: upregulated; blue: downregulated in the old group.

To identify biological processes associated with aging, a GO BP enrichment analysis was performed independently on the DEGs identified in bone marrow (Table S7) and blood (Table S8). For bone marrow, the top 15 most enriched biological processes included those related to immune response and inflammation (GO:0050729, GO:0050727, GO:0002429, GO:0002768, and GO:0002274), cell migration and chemotaxis (GO:0050900, GO:0006935, and GO:0042330), and glial cell differentiation or nervous system development (GO:0010001, GO:0048708, GO:0042063, GO:0048710, and GO:0051960), as well as muscle cell proliferation (GO:0033002) and positive regulation of the MAPK cascade (GO:0043410) (Figure 1A).

Among the genes involved in these biological processes, the most frequently encountered were proinflammatory cytokines (*IL6* and *TNF*), Toll-like receptors (*TLR1*, *TLR2*, *TLR4*, and *TLR6*), chemokines and their receptors (*CCL5*, *CCL3*, *CCR7*, and *CXCR1*), chemotaxis receptors (*C5AR1*, *FPR1*, and *FPR2*), myeloid cell markers (*TREM2*, *CD177*, and *LRRK2*), Fc receptors (*FCGR1A* and *FCGR2B*), and the signaling protein *STAT3*.

The top 15 biological processes for peripheral blood represented functional categories such as immune response-related cell surface receptor signaling (GO:0002768 and GO:0002429), T-cell differentiation and activation (GO:0030217, GO:0030098, GO:0046631, GO:0046632, and GO:0050863), regulation of innate immunity (GO:0002274, GO:0045089, GO:0002833, and GO:0071216), leukocyte cell–cell adhesion (GO:0007159 and GO:1903037), leukocyte migration (GO:0050900), and regulation of the immune effector process (GO:0002697) (Figure 1B).

These GO terms were predominantly represented by T-cell receptor components and signaling molecules (*CD3D*, *CD3E*, *CD3G*, *CD8A*, *ZAP70*, *LCK*, *LAT*, *ITK*, *PTPN22*, *RASGRP1*, and *VAV1*), Toll-like receptors (*TLR1*, *TLR2*, *TLR4*, *TLR5*, *TLR6*, and *TLR10*) and their co-receptors (*CD14* and *LY96*), Fc receptors (*FCGR2B* and *FCAR*), chemotaxis receptors (*C5AR1*, *C5AR2*, *FPR1*, *FPR2*, and *FPR3*), myeloid cell markers (*TREM2* and *CD177*), protein kinases (*SYK*, *HCK*, *LYN*, and *FYN*), transcription factors and signaling proteins (*SPI1*, *STAT3*, *STAT4*, *STAT5B*, *TBX21*, *ZBTB16*, and *TOX*), effector molecules (*IFNG*, *CD274*, *IDO1*, and *IL23A*), and cell cycle and metabolic regulators (*ADA* and *CDKN2A*).

To visualize the relationships between enriched biological processes, enrichment maps (emapplots) were constructed for the top 20 most significant processes for the bone marrow and peripheral blood. The resulting networks demonstrate functional clustering of terms in the analyzed tissues.

In the bone marrow, enriched terms are organized into several functional clusters. The largest cluster (1) groups terms associated with the innate immune response and the regulation of the nervous system, which may indicate neuroimmune interactions in the bone marrow microenvironment. Separate small clusters are formed by terms related to leukocyte migration and chemotaxis (2), cell proliferation (3), and the immune response mediated by cell surface receptors (4). An isolated cluster is formed by the term “positive regulation of MAPK cascade” (5) (Figure 1C).

In the peripheral blood, most of the enriched terms formed a single, large network (1) combining terms related to both the innate and adaptive immune responses, suggesting a more integrated regulation of immune processes in blood. In addition to the main network, two secondary clusters were identified: response to biotic stimulus (2) and myeloid leukocyte activation (3) (Figure 1D).

To determine the global transcriptional shifts across the entire expression profile, GSEA was conducted using the GO BP and KEGG databases (Tables S9–S12). GSEA revealed that biological processes and signaling pathways associated with immune functions were predominantly negatively enriched (NES < 0) in both bone marrow and blood.

Downregulated GO terms in the bone marrow included positive regulation of defense response (normalized enrichment score (NES) = −1.81), cytokine-mediated signaling pathway (NES = −1.76), myeloid leukocyte migration (NES = −1.94), myeloid leukocyte activation (NES = −1.82), cellular response to biotic stimulus (NES = −1.85), and regulation of the inflammatory response (NES = −1.80). The KEGG pathways downregulated in this tissue encompassed the hematopoietic cell lineage (NES = −2.03), cytokine–cytokine receptor interaction (NES = −1.84), chemokine signaling pathway (NES = −1.72), viral protein interaction with cytokine and cytokine receptor (NES = −1.80), Toll-like receptor signaling pathway (NES = −1.77), osteoclast differentiation (NES = −1.67), FoxO signaling pathway (NES = −1.60), NOD-like receptor signaling pathway (NES = −1.58), and C-type lectin receptor signaling pathway (NES = −1.60) (Figure 1E).

Attenuation of immune processes was also observed in the peripheral blood. The downregulated GO terms included phagocytosis (NES = −1.88), response to bacterium (NES = −1.69), myeloid leukocyte migration (NES = −1.91), positive regulation of defense response (NES = −1.68), cytokine-mediated signaling pathway (NES = −1.59), activation of the innate immune response (NES = −1.62), and regulation of the inflammatory response (NES = −1.72), whereas the enriched KEGG pathways spanned the chemokine signaling pathway (NES = −1.77), Toll-like receptor signaling pathway (NES = −1.70), NOD-like receptor signaling pathway (NES = −1.82), C-type lectin receptor signaling pathway (NES = −1.58) and B-cell receptor signaling pathway (NES = −1.67). In addition, a number of upregulated immune-related pathways were identified in the peripheral blood, such as the T-cell receptor signaling pathway (NES = 1.67), primary immunodeficiency (NES = 1.78), allograft rejection (NES = 1.77), and natural killer cell-mediated cytotoxicity (NES = 1.56) (Figure 1F).

### 3.2. Shared Biological Pathways and Expression Patterns in Bone Marrow and Blood

**Figure 2:**
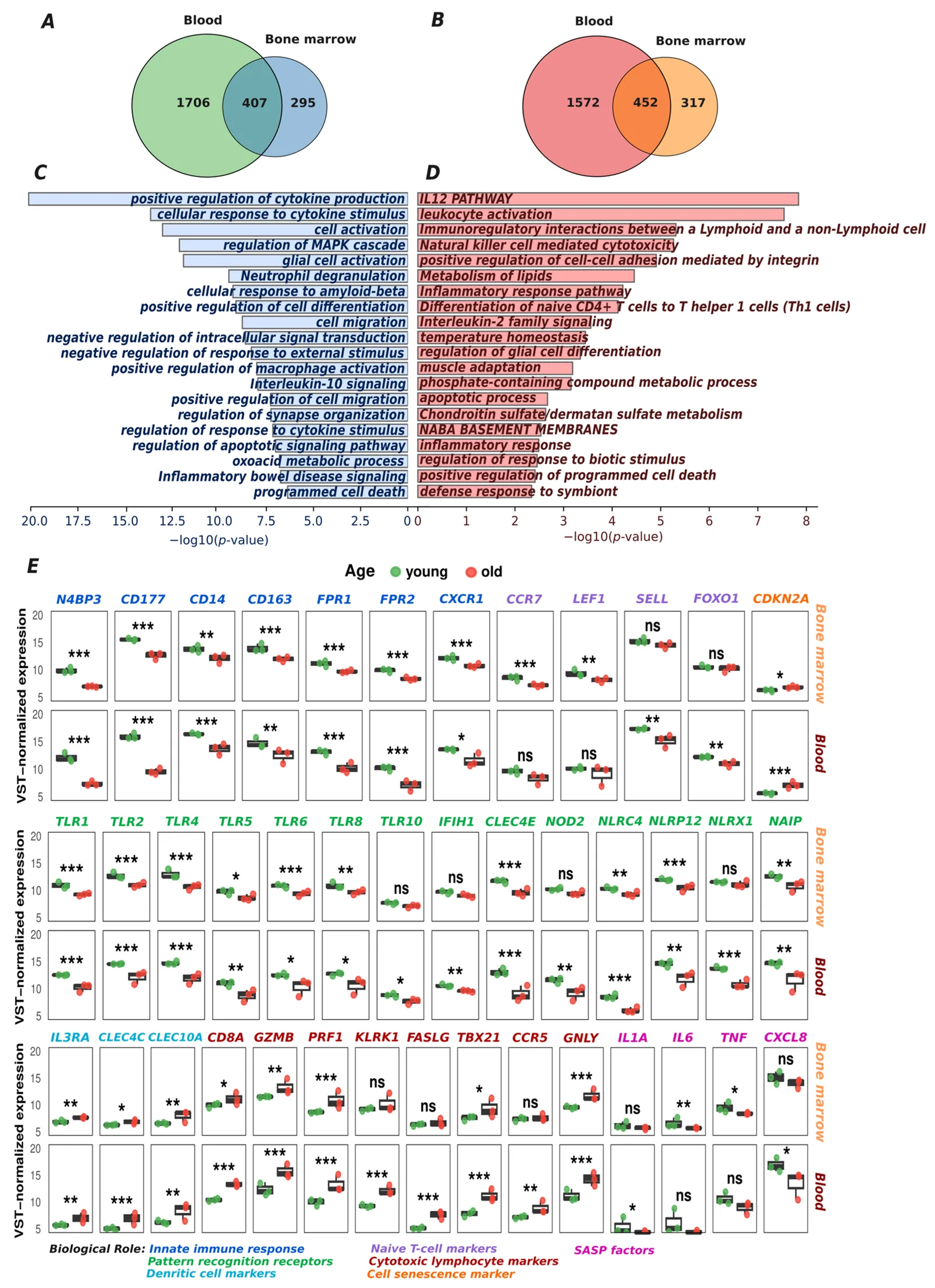
(**A**,**B**) Venn diagrams showing the intersection of downregulated (**A**) and upregulated (**B**) DEGs in the aged cynomolgus macaques between bone marrow and blood. (**C**,**D**) Functional enrichment analysis of downregulated (**C**) and upregulated genes (**D**) overlapping between bone marrow and blood. The x-axis displays the negative log-transformed *p*-values (−log10(*p*-value)) for the enriched biological terms; longer bars indicate higher statistical significance. (**E**) Box plots of immune-related gene expression in bone marrow and peripheral blood of young (*n* = 3, green) and old (*n* = 3, red) cynomolgus macaques. The y-axis represents DESeq2 VST-normalized gene expression. Statistical significance: * *p*.adj < 0.05, ** *p*.adj < 0.01, *** *p*.adj < 0.001, ns: not significant.

The intersection of DEGs between bone marrow and blood revealed 407 overlapping downregulated genes (Table S13, Figure 2A) and 452 overlapping upregulated genes in the aged cynomolgus macaques (Table S14, Figure 2B). The presence of these shared expression patterns underlines a conserved systemic transcriptomic response to aging that bridges the bone marrow and peripheral circulation.

Functional enrichment analysis was performed on the overlapping down- and upregulated genes using the Metascape platform, which integrates information from multiple bioinformatics databases. Among the downregulated genes, the most significant enrichments were observed in inflammatory and innate immune signaling cascades, including positive regulation of cytokine production (GO:0001819), cellular response to cytokine stimulus (GO:0071345), regulation of MAPK cascade (GO:0043408), neutrophil degranulation (R-HAS-6798695), macrophage activation (GO:0043032), and cell migration (GO:0016477 and GO:0030335) (Figure 2C).

Among the upregulated genes, pathways associated with the adaptive immune response were significantly enriched, including naive CD4+ T-cell differentiation into Th1 effectors (R-HSA-9942503) and interleukin-2 signaling (R-HSA-451927). Enrichment was also observed for innate immune processes: activation of NK-mediated cytotoxicity (KEGG:hsa04650) and the inflammatory response (GO:0006954 and WP453). In addition to immune functions, metabolic processes were enriched: lipid metabolism (R-HSA-556833) and glycosaminoglycan metabolism (R-HSA-1793185) (Figure 2D).

Interestingly, apoptosis-associated terms were present among both upregulated (GO:0006915 and GO:0043068) and downregulated (GO:2001233 and GO:0012501) DEGs.

Among the most significant genes with reduced expression in old cynomolgus macaques in both bone marrow and blood were markers of the innate immune response: *N4BP3*, a positive regulator of the response to viral infections, and *CD177*, a surface marker of neutrophils responsible for their migration to sites of inflammation. The expression of monocyte markers *CD14* and *CD163* also decreased similarly in both tissues. Reduced expression in the old group was also detected for the *FPR1* and *FPR2* genes, encoding receptors expressed on the surface of a wide range of innate immune cells, predominantly neutrophils, monocytes, and macrophages, as well as eosinophils and NK cells. Additionally, we observed decreased expression of *CXCR1*, a chemokine receptor critical for neutrophil chemotaxis, and its ligand *CXCL8* (IL-8), which together mediate neutrophil recruitment to sites of inflammation (Table S15, Figure 2E).

Analysis of genes with the same direction of expression changes in both tissues revealed decreased expression of most Toll-like receptor (TLR) family genes in the old cynomolgus macaque group (*TLR1*, *TLR2*, *TLR4*, *TLR5*, *TLR6*, and *TLR8*). The expression of *TLR10* was also reduced, but statistical significance was achieved only in the peripheral blood. To further investigate whether the observed decline in TLR expression reflects a broader suppression of pattern-recognition receptors (PRRs), we examined the expression of key genes encoding the RLR (RIG-I-like receptor), CLR (C-type lectin receptor) and NOD-like receptor (NLR) families. Regarding the RLR family, *IFIH1* (MDA5) was significantly downregulated with age in the blood, with a similar trend in the bone marrow, while *RIGI* (DDX58) and *DHX58* (LGP2) showed no significant changes. Among the CLRs, *CLEC4E* (Mincle) showed a marked and significant decrease in both blood and bone marrow, whereas *CLEC7A* (Dectin-1), *CLEC6A* (Dectin-2), and *CLEC9A* (DNGR-1) remained largely unchanged. Within the NLR family, *NOD2*, *NLRC4*, *NLRP12*, and *NAIP* displayed a consistent decline in expression across both blood and bone marrow, whereas the downregulation of *NLRX1* was confined solely to the blood (Table S15, Figure 2E).

To extend our analysis of the myeloid compartment, we examined the expression of DC lineage markers. In both blood and bone marrow, we observed significant upregulation of pDC markers *IL3RA* and *CLEC4C* and the cDC2 marker *CLEC10A*, while other DC markers, including *CLEC9A*, *XCR1*, *CADM1*, *CD1C*, and *FCER1A*, exhibited no significant changes (Table S15, Figure 2E).

Conversely, the cytotoxic T-cell marker *CD8A* increased in expression with age in both tissues. Also, cytotoxic lymphocyte markers showed increased expression primarily in the peripheral blood: *GZMB*, *PRF1*, *KLRK1*, *FASLG*, *TBX21*, *CCR5*, and *GNLY*. However, in the bone marrow, only *GZMB*, *PRF1*, *TBX21*, and *GNLY* exceeded the significance threshold. In contrast, several naive T-cell markers decreased in expression: *CCR7* and *LEF1* in the bone marrow and *SELL* and *FOXO1* in the peripheral blood. Increased expression of the cell cycle regulator and the senescent cell marker *CDKN2A* (p16) was also detected in both tissues. By contrast, another canonical senescence marker, *CDKN1A* (p21), did not show significant age-associated changes in either tissue. Genes encoding key proinflammatory cytokines and chemokines, *IL1A*, *IL1B*, *IL6*, *TNF*, *CXCL8*, and *CCL2*, were also analyzed. *IL6* and *TNF* expression was significantly reduced only in the bone marrow, while *IL1A* and *CXCL8* expression was decreased only in the peripheral blood (Table S15, Figure 2E).

## 4. Discussion

The substantially higher number of DEGs in the peripheral blood (4137) compared to the bone marrow (1471) may reflect the systemic nature of blood as a compartment that integrates age-related inflammatory and metabolic signals from the entire organism. In contrast, the bone marrow niche, with its specialized stromal microenvironment, may partially buffer hematopoietic cells from systemic perturbations, resulting in a more restricted transcriptional response. Furthermore, the greater cellular heterogeneity of blood likely contributes to a broader spectrum of age-associated transcriptional changes. This tissue-specific difference highlights blood as a more sensitive indicator of systemic immunosenescence.

We observed similar patterns of age-related changes in the immune system of cynomolgus macaques across both bone marrow and peripheral blood. Previous studies have documented age-related depletion of naive T cells in the peripheral blood of both macaques and humans, highlighting this as a conserved hallmark of immunosenescence across species [56,57]. Notably, our analysis revealed a significant decrease in the naive T-cell markers *CCR7* and *LEF1* in the bone marrow, along with a concurrent decline in *SELL* and *FOXO1* in the peripheral blood.

Pathway enrichment analysis of overlapping DEGs between blood and bone marrow revealed a coordinated immune signature, highlighted by naive CD4+ T-cell differentiation into Th1 effectors, interleukin-2 signaling, and activation of NK-mediated cytotoxicity. Consistent with these pathway annotations, we detected increased expression of markers associated with cytotoxic lymphocyte activation, including *GZMB*, *PRF1*, *KLRK1*, *FASLG*, *TBX21*, *CCR5*, and *GNLY*, suggesting enhanced activity in aged animals. The accumulation of CD8+ T cells exhibiting an NK-like phenotype is a recognized hallmark of immune aging in primate blood, including humans [8,58]. These findings align with the concept of T-cell reprogramming, wherein terminally differentiated, senescent CD8+ T cells lose their classical adaptive properties and acquire features characteristic of innate immune cells [58,59].

One of the most interesting effects of aging identified in our study is the decreased expression of myeloid cell markers associated with neutrophils and monocytes (*CD177*, *CD14*, *CD163*, *CXCR1*, *FPR1* and *FPR2*). *FPR1* and *FPR2* are essential for bacterial ligand recognition. These receptors, along with *CD177*, collectively mediate neutrophil activation and directed chemotaxis. Decreased expression of these genes indicates an age-related decline in neutrophil sensitivity to pathogens, diminished functional activity [27,33], and a disruption of the signaling cascades that mediate directed cell migration [27,60]. The simultaneous downregulation of *CXCR1* and its ligand *CXCL8* (IL-8) is consistent with an age-associated decline in neutrophil chemotactic capacity, as CXCR1/CXCL8 signaling is essential for neutrophil recruitment to sites of infection [61]. Highlighting the scale of these transcriptional changes, GSEA in both tissues confirmed a robust downregulation of pathways broadly related to myeloid cell functions, including cell activation, migration, chemotaxis, and the response to biotic or bacterial stimuli. This age-related transcriptional remodeling of the myeloid compartment may be driven by reduced cell frequency, altered cell-intrinsic gene expression, or a combination of both.

More specifically, this systemic, age-associated suppression heavily targeted innate immune sensing. GSEA demonstrated that the TLR, NLR, and CLR signaling pathways were all significantly downregulated in both blood and bone marrow. Consistent with these pathway alterations, we observed a broad and significant downregulation of individual PRR genes across multiple families. This included a large panel of TLRs (*TLR1*, *TLR2*, *TLR4*, *TLR5*, *TLR6*, *TLR8*, and *TLR10*), the RLR sensor *IFIH1* (MDA5), the CLR member *CLEC4E* (Mincle), and several NLR family members (*NOD2*, *NLRC4*, *NLRP12*, and *NAIP*). Together with the reduced expression of the classical monocyte marker *CD14* (a co-receptor of the TLR4 pattern-recognition complex) and the anti-inflammatory macrophage marker *CD163*, these findings point to a broad innate immune dysregulation, consistent with reduced responsiveness to infectious stimuli [28,29,62].

The interpretation of the DC compartment transcriptomic changes remains challenging, as the marked upregulation of *IL3RA*, *CLEC4C*, and *CLEC10A* is accompanied by a lack of significant changes in other key lineage markers, including *CD1C*, *FCER1A*, *CLEC9A*, *XCR1*, and *CADM1*. Because DCs constitute an extremely small fraction of total myeloid cells, their transcriptomic shifts can easily become blurred without targeted cell enrichment or larger cohorts. Future studies utilizing flow cytometry, cell sorting, or single-cell sequencing will be essential to overcome these sample limitations and define specific cell-type contributions.

Importantly, our study did not detect an increase in classical SASP-related proinflammatory cytokines (e.g., *IL1A*, *IL1B*, *IL6*, *TNF*, *CXCL8*, and *CCL2*). While this may appear to contradict the established paradigm of age-related “sterile” chronic low-grade inflammation, it suggests that age-associated systemic inflammation may be maintained not by an excess of interleukins but rather by chronic cytotoxic aggression from activated T cells and NK cells. This finding is further supported by large-scale transcriptomic atlases of immunosenescence in individuals of extreme old age [63], which are consistent with our data on increased expression of cytotoxic lymphocyte markers in the aged cynomolgus macaques.

At the same time, the age-related increase in the expression of *CDKN2A* (p16), an inhibitor of cyclin-dependent kinases and a key marker of cellular senescence, may indicate the accumulation of senescent cells in the studied tissues, which is a sign of their chronological and functional aging [64,65]. By contrast, *CDKN1A* (p21) showed no significant age-associated changes in either blood or bone marrow. This is consistent with the notion that p21 is more closely associated with acute stress responses, whereas p16 is a marker of chronic, persistent senescence in aging tissues [66].

Widespread activation of the cytotoxic machinery in the bone marrow and peripheral blood points to an emerging autoimmune-like phenotype. While the *PRF1/GZMB* axis is vital for eliminating virally infected or neoplastic cells, its regulation appears to become less precise with age [67]. This loss of specificity is further underscored by the overexpression of *GNLY* and the stress-sensing receptor *KLRK1* (*NKG2D*), both of which may inadvertently promote tissue damage when misdirected toward host cells [68,69].

Furthermore, increased *FASLG* expression triggers apoptosis by binding to the Fas receptor; however, its persistent overexpression in the blood of aged animals may paradoxically provoke hyperinflammation and immune exhaustion [70]. Similarly, the upregulated expression of *TBX21* (T-bet)—a master regulator of cytotoxic lymphocyte development and maturation—alongside increased *CCR5* (a chemokine receptor that directs immune cells to sites of tissue inflammation) further supports the hypothesis of autoimmune-like inflammation in aged macaques.

This heightened cytotoxic profile likely stems from either an underlying autoimmune-like disorder or a compensatory, hyperreactive response to the exhaustion of adaptive immunity. Furthermore, the observed downregulation of *CD163* and *FPR2*, key markers of anti-inflammatory M2 macrophages—which are typically responsible for suppressing T-cell activity and facilitating tissue repair—appears to diminish the immune system’s capacity to regulate this cytotoxic response.

## 5. Conclusions

In summary, the present transcriptomic analysis identified age-associated immune changes in the bone marrow and peripheral blood of cynomolgus macaques. Notably, the findings did not support the presence of an age-related inflammatory signature typically associated with the senescence-associated secretory phenotype (SASP). Instead, the aging immunotranscriptome was characterized by an enhanced lymphocyte cytotoxic response, diminished functional capacity of anti-inflammatory and tissue-reparative myeloid cells, and impaired recognition of bacterial molecular patterns. These transcriptomic signatures provide new insights into the mechanisms underlying age-related immune dysregulation in non-human primates and establish a preliminary framework for prioritizing candidate genes for future molecular and functional validation studies.

## 6. Limitations

This study has several limitations. First, the small sample size (*n* = 3 per age group) may have limited the statistical power to detect differential expression for individual genes. This cohort size was dictated by ethical considerations regarding the use of non-human primates in biomedical research. Second, because of the limited cohort size, only male animals were included to minimize biological variability and eliminate sex as a potential confounding factor. Third, the study population had relatively low genetic diversity. A colony of cynomolgus macaques (*M. fascicularis*) has been maintained at the primatology center under inbred breeding conditions for approximately 35 years (5–6 generations). Although this breeding strategy reduces genetic diversity, it provides a more homogeneous genetic background than populations recently captured from the wild or obtained from large commercial breeding facilities. To partially mitigate this limitation, animals were selected from different family lineages that had not been interbred for at least two generations. Fourth, because only male animals from a genetically homogeneous colony were included, our findings may not fully capture the sex-specific and genetically driven features of immunosenescence. As this is a pilot study designed to identify the major transcriptomic changes associated with aging, future investigations should include larger cohorts representing both sexes and greater genetic diversity. Fifth, this study focused exclusively on the transcriptomic analysis of bone marrow and peripheral blood cells. Consequently, poly(A)-enriched mRNA was used for library preparation, and non-polyadenylated transcripts, including many classes of non-coding RNAs, were not examined. Although this limitation does not affect the interpretation of differential expression analyses of protein-coding genes, future whole-transcriptome studies incorporating small RNA and other non-coding RNA analyses may provide additional insights into the regulatory mechanisms underlying immunoaging. Sixth, because this study represents a preliminary transcriptomic screen, the identified immunosenescence-associated markers should be considered provisional and require validation using independent molecular, cellular, and functional approaches.

## Data Availability

Raw data of this study are available from the corresponding author upon reasonable request.

## Supplementary Materials

The following supporting information can be downloaded at https://www.mdpi.com/article/10.3390/genes17080944/s1, Figure S1: Bone marrow aspiration procedure from the upper third of the macaque tibial shaft; Figure S2: (A,B) Volcano plots showing statistically significant DEGs in bone marrow (A) and blood (B). (C,D) Heatmaps of the top 50 DEGs ranked by significance (*p*.adj) in bone marrow (C) and blood (D). (E,F) PCA plots of gene expression profiles in bone marrow (E) and blood (F); Figure S3: Additional PCA plots of bone marrow and blood samples; Table S1: Individual data of animals involved in the experiments; Table S2: RNA-seq quality control metrics; Table S3: Upregulated DEGs in bone marrow; Table S4: Downregulated DEGs in bone marrow; Table S5: Upregulated DEGs in blood; Table S6: Downregulated DEGs in blood; Table S7: GO Biological Process enrichment analysis of DEGs in bone marrow; Table S8: GO Biological Process enrichment analysis of DEGs in blood; Table S9: GSEA results of GO Biological Processes in bone marrow; Table S10: GSEA results of KEGG pathways in bone marrow; Table S11: GSEA results of GO Biological Processes in blood; Table S12: GSEA results of KEGG pathways in blood; Table S13: Downregulated DEGs overlapping between bone marrow and blood; Table S14: Upregulated DEGs overlapping between bone marrow and blood; Table S15: Immune-related genes with age-associated expression changes in bone marrow and blood.

## Abbreviations

The following abbreviations are used in this manuscript:

SASP	senescence-associated secretory phenotype
TCR	T-cell receptor
BCR	B-cell receptor
PRR	pattern-recognition receptor
TLR	Toll-like receptor
RLR	RIG-I-like receptor
CLR	C-type lectin receptor
NLR	NOD-like receptor
DC	dendritic cell
PAMPs	pathogen-associated molecular patterns
HSPC	hematopoietic stem and progenitor cell
RIN	RNA integrity number
DEGs	differentially expressed genes
*p*.adj	Benjamini–Hochberg adjusted *p*-value
GO	gene ontology
BP	biological process
KEGG	Kyoto Encyclopedia of Genes and Genomes
GSEA	gene set enrichment analysis
NES	normalized enrichment score
