# Supplementary material for: Transcriptomic Markers of Immunosenescence in Cynomolgus Macaques: A Pilot Study
Source: Genes (Basel). 2026 Aug 13;17(8):944. doi: 10.3390/genes17080944 (PMC13511921; doi:10.3390/genes17080944)
Supplement: Supplementary file 1 [file genes-17-00944-s001.zip › Supplementary material.proof/Supplementary_Figures.pdf]

Supplementary Figures

Transcriptomic Markers of Immunosenescence in Cynomolgus Macaques: A Pilot Study

Authors: Viktoria M. Petrova, Dmitry V. Bulgin, Elena Yu. Radomskaya, Vsevolod A. Shevelov, Darya S. Zhukova, Olga. P. Chzhu, Andrey D. Manakhov, Aleksander V. Popov, Stanislav A. Rybtsov*

Figure S1. Bone marrow aspiration procedure from the upper third of the macaque tibial shaft.

Figure S2. (**A, B**) Volcano plots showing statistically significant DEGs in bone marrow (**A**) and blood (**B**). (**C, D**) Heatmaps of the top 50 DEGs ranked by significance (*p*.adj) in bone marrow (**C**) and blood (**D**). (**E, F**) PCA plots of gene expression profiles in bone marrow (**E**) and blood (**F**).

Figure S3. Additional PCA plots of bone marrow and blood samples.

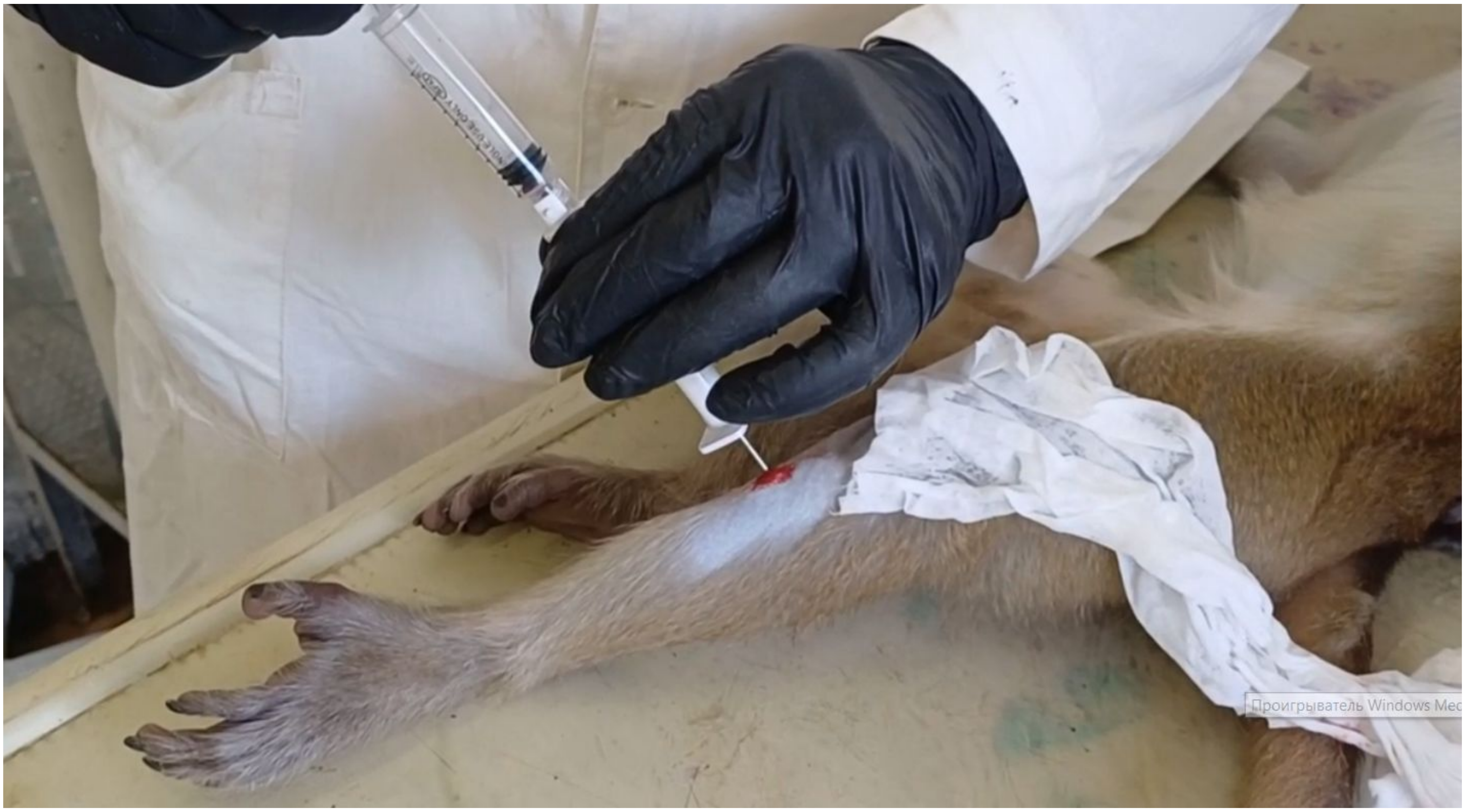

Figure S1. Bone marrow aspiration procedure from the upper third of the macaque tibial shaft.

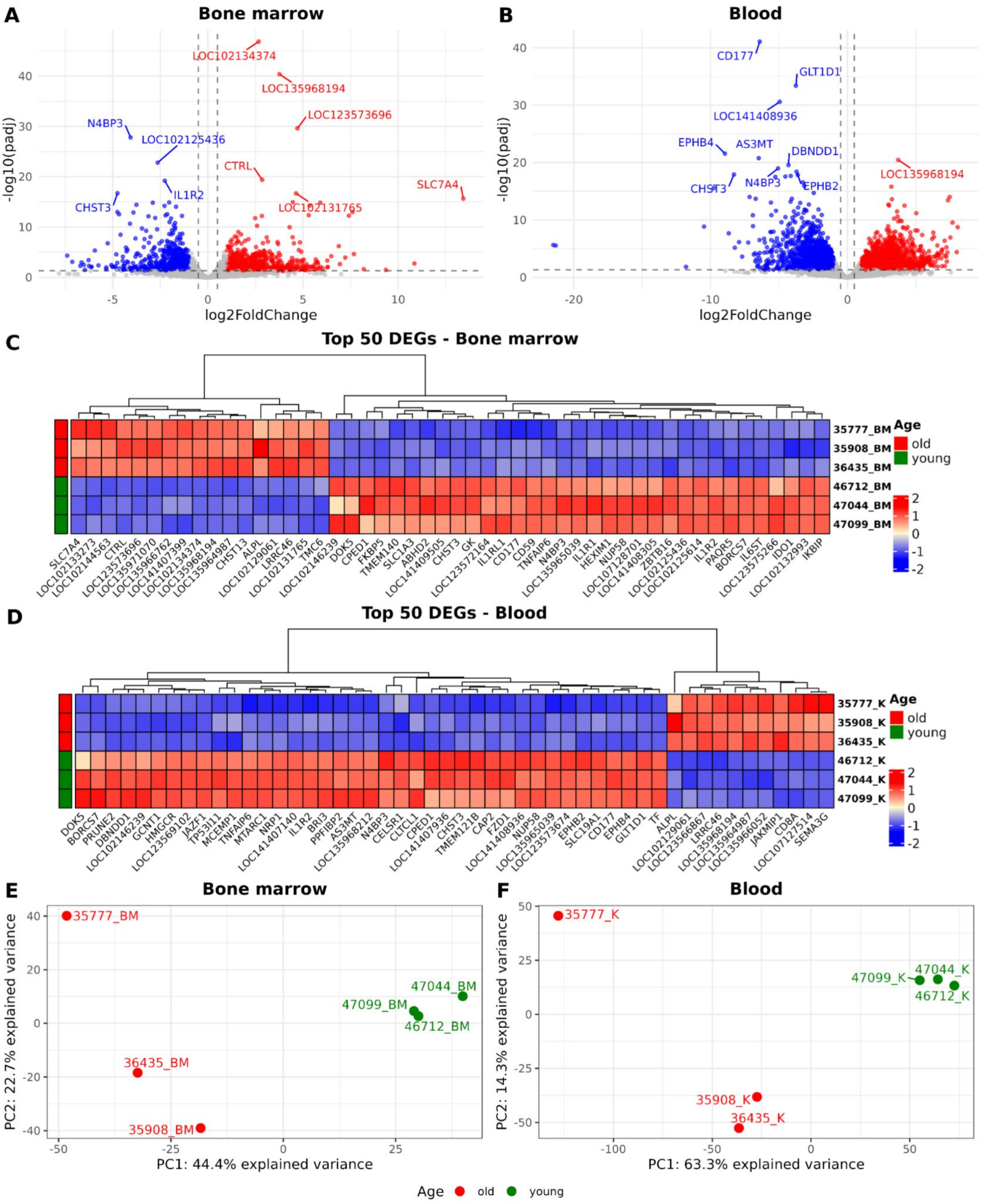

Figure S2. (**A, B**) Volcano plots showing statistically significant DEGs in bone marrow (**A**) and blood (**B**). Up-regulated genes (log2FC > 1, *p*.adj < 0.05) are highlighted in red; down-regulated genes (log2FC < −1, *p*.adj < 0.05) are in blue. (**C, D**) Heatmaps of the top 50 DEGs ranked by significance (padj) in bone marrow (**C**) and blood (**D**). Animal groups are indicated by color bars: young (green) and old (red). The heatmap color scale represents relative expression levels (red: up-regulated, blue: down-regulated). (**E, F**) PCA plots of gene expression profiles in bone marrow (**E**) and blood (**F**). Samples are colored by age (green: young; red: old). Axes display the percentage of variance explained by the first (PC1) and the second (PC2) principal components.

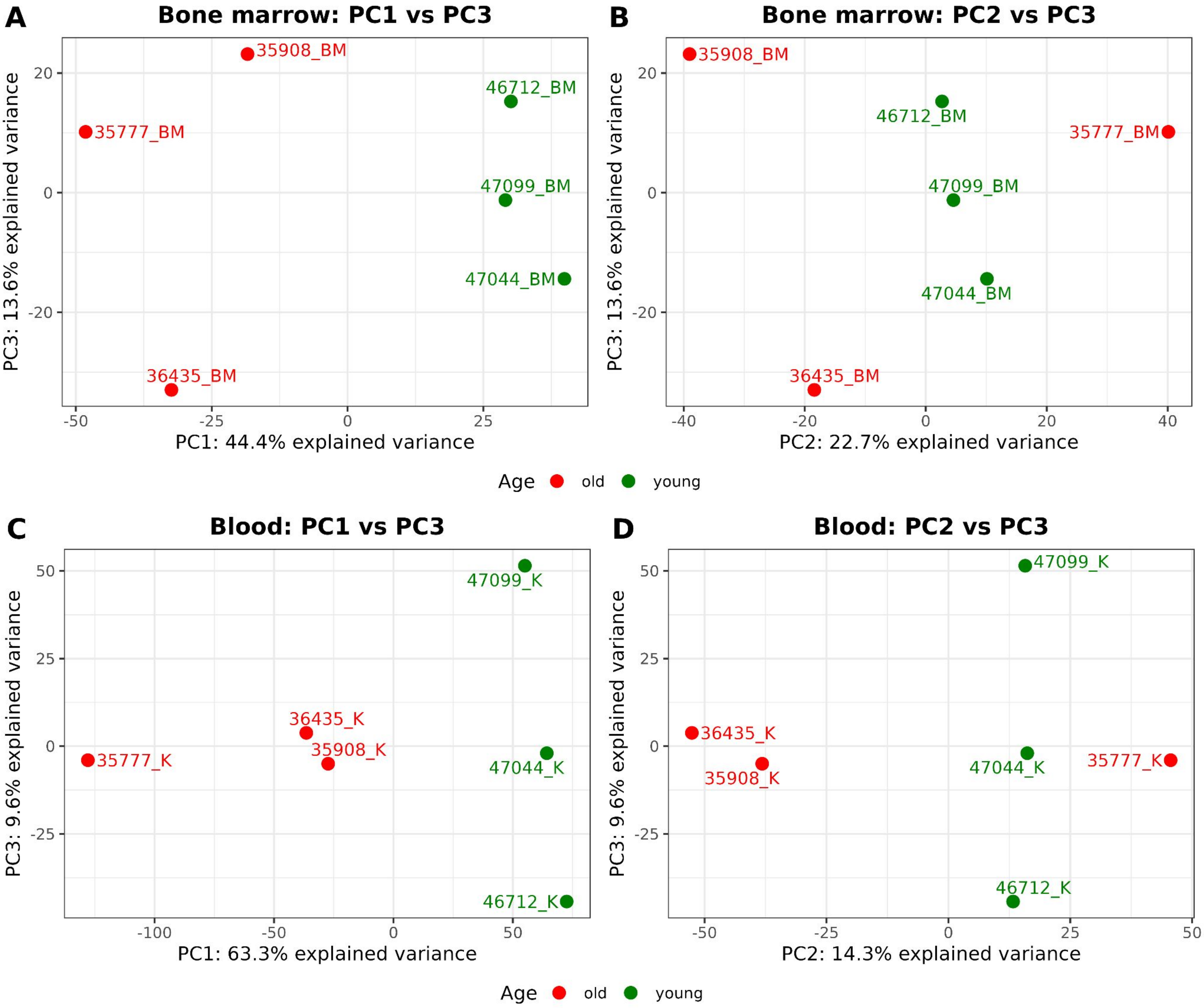

Figure S3. Additional PCA plots of bone marrow and blood samples. Panels show PC1 vs PC3 for bone marrow (**A**), PC2 vs PC3 for bone marrow (**B**), PC1 vs PC3 for blood (**C**), and PC2 vs PC3 for blood (**D**). Samples are colored by age (green: young; red: old). The percentages of variance explained by each principal component are indicated on the axes.
